# Correction: BRAF^V600E^-Driven Lung Adenocarcinoma Requires Copper to Sustain Autophagic Signaling and Processing

**DOI:** 10.1158/1541-7786.MCR-26-0652

**Published:** 2026-10-02

**Authors:** Tiffany Tsang, Xingxing Gu, Caroline I. Davis, Jessica M. Posimo, Zoey A. Miller, Donita C. Brady

In the original version of this article (1), Fig. 3C contained two sets of three duplicated crystal violet [bromocresol purple (BCP)] image panels, wherein the same panels represented both group #25 and #27. The authors stated that the panels are correct for group #27, incorrect for group #25, and provided the intended panels for group #25. The error has been corrected in the latest online HTML and PDF versions of the article. The authors regret this error.
